# Muscle acellular scaffold as a biomaterial: effects on C2C12 cell differentiation and interaction with the murine host environment

**DOI:** 10.3389/fphys.2014.00354

**Published:** 2014-09-26

**Authors:** Barbara Perniconi, Dario Coletti, Paola Aulino, Alessandra Costa, Paola Aprile, Luigi Santacroce, Ernesto Chiaravalloti, Laura Coquelin, Nathalie Chevallier, Laura Teodori, Sergio Adamo, Massimo Marrelli, Marco Tatullo

**Affiliations:** ^1^Department of Biological Adaptation and Aging (B2A) UMR 8256 CNRS - ERL U1164 INSERM, Sorbonne Universités, UPMC University Paris 06Paris, France; ^2^Maxillofacial Unit, Calabrodental ClinicCrotone, Italy; ^3^AHFOS Department - Section of Histology and Medical Embryology, Sapienza University of RomeRome, Italy; ^4^Interuniversitary Institute of Miology (IIM)Rome, Italy; ^5^UTAPRAD-DIM, ENEAFrascati, Italy; ^6^Tor Vergata University of RomeRome, Italy; ^7^JSGEM Department - Section of Taranto, University of BariTaranto, Italy; ^8^Unite d'Ingénierie et de Therapie Cellulaire, Etablissement Français du Sang Ile de France, Université Paris-Est CréteilCréteil, France; ^9^Regenerative Medicine Section, Tecnologica Research InstituteCrotone, Italy

**Keywords:** extracellular matrix, niche, osteogenic differentiation, myogenic differentiation, tissue engineering, regenerative medicine

## Abstract

The extracellular matrix (ECM) of decellularized organs possesses the characteristics of the ideal tissue-engineering scaffold (i.e., histocompatibility, porosity, degradability, non-toxicity). We previously observed that the muscle acellular scaffold (MAS) is a pro-myogenic environment *in vivo*. In order to determine whether MAS, which is basically muscle ECM, behaves as a myogenic environment, regardless of its location, we analyzed MAS interaction with both muscle and non-muscle cells and tissues, to assess the effects of MAS on cell differentiation. Bone morphogenetic protein treatment of C2C12 cells cultured within MAS induced osteogenic differentiation *in vitro*, thus suggesting that MAS does not irreversibly commit cells to myogenesis. *In vivo* MAS supported formation of nascent muscle fibers when replacing a muscle (orthotopic position). However, heterotopically grafted MAS did not give rise to muscle fibers when transplanted within the renal capsule. Also, no muscle formation was observed when MAS was transplanted under the xiphoid process, in spite of the abundant presence of cells migrating along the laminin-based MAS structure. Taken together, our results suggest that MAS itself is not sufficient to induce myogenic differentiation. It is likely that the pro-myogenic environment of MAS is not strictly related to the intrinsic properties of the muscle scaffold (e.g., specific muscle ECM proteins). Indeed, it is more likely that myogenic stem cells colonizing MAS recognize a muscle environment that ultimately allows terminal myogenic differentiation. In conclusion, MAS may represent a suitable environment for muscle and non-muscle 3D constructs characterized by a highly organized structure whose relative stability promotes integration with the surrounding tissues. Our work highlights the plasticity of MAS, suggesting that it may be possible to consider MAS for a wider range of tissue engineering applications than the mere replacement of volumetric muscle loss.

## Introduction

A niche is composed of elements that surround stem cells: tissue specific cells, extracellular matrix and local growth factors (Yin et al., 2013). These elements determine the local microenvironment that supports the maintenance of stem cell identity and regulates the function of stem cells (Kuang et al., 2008). In addition, the niche supports stem cells and controls their self-renewal *in vivo* (Spradling et al., 2001) by modulating the asymmetric cell division insomuch as it ensures stem cell renewal and production of a sufficient number of committed daughter cells for tissue homeostasis and repair (Kuang et al., 2008). It is worth bearing in mind that the local microenvironment affects not only stem cell behavior (particularly the stem cell specific auto-renewal feature) but also the differentiation potential and cell division of committed daughter cells deriving from stem cell asymmetrical division. Indeed, a fibroblast-specific niche has been described for cell culture purposes (Sivan et al., 2014), while the bone marrow niche that regulates hematopoietic stem cells is also reported to be necessary for B-cell commitment (Adler et al., 2014). Future challenges involved in the recreation of cell niches as platforms for culture models, which will allow the true *in situ* regenerative niche to be investigated, have been reviewed by Kirkpatrick (Kirkpatrick, 2014).

The definition of the microenvironment affecting both stem cell renewal and committed daughter cell differentiation is of particular relevance to tissue engineering (TE). TE represents an innovative approach based on the emulation of neo-organogenesis aimed at recreating a wide range of tissues to be used to replace lost tissues (Klumpp et al., 2010). A commonly applied definition of TE, provided by Langer and Vacanti, is “an interdisciplinary field that applies the principles of engineering and life sciences toward the development of biological substitutes that restore, maintain, or improve tissue function or a whole organ” (Langer and Vacanti, 1993). For TE purposes cells are often transplanted or seeded into a structure capable of supporting three-dimensional tissue formation. These structures, referred to as scaffolds, are critical, both *ex vivo* and *in vivo*, to recapitulate the niche and to support cell adhesion, survival and differentiation. Indeed, not only do scaffolds allow cell migration and/or attachment, but they also deliver and retain cells and biochemical factors, permit the diffusion of vital cell nutrients and exert mechanical and biological influences that control cell behavior (Macchiarini et al., 2008; Whitney et al., 2012). Scaffolds may be made of either natural or synthetic materials. Indeed, various derivatives of the extra cellular matrix (ECM) have been studied because they possess all the features of the ideal tissue-engineered scaffold or biomaterial, which include histocompatibility, bioactivity, porosity, degradability, non-toxicity and mechanical properties that match those of the original tissue (Borschel et al., 2004).

An alternative to the production of constructs composed of cells seeded into scaffolds is ECM. ECM can be used to support *in situ* regeneration, thereby relying on the bioactivity of autologous or heterologous biomaterial on autologous cells. Indeed, ECM is manufactured by the resident cells of each tissue and organ and is in a state of dynamic equilibrium with its surrounding microenvironment. We may assume, even without deciphering the complex three-dimensional organization of the structural and functional molecules of which it is composed, that ECM is biocompatible because cells produce their own matrix (Badylak, 2007). Recently, an increasing amount of attention has been paid to the use of ECM-based scaffolds for TE interventions. ECM-based scaffolds not only preserve the structure and molecular features of the native ECM, but also release matricryptic peptides during degradation. Matricryptic peptides affect cell motility, proliferation and differentiation, thereby greatly influencing the constructive remodeling of new tissue (Faulk et al., 2013). For these reasons, various forms of intact ECM have been used as biological scaffolds to promote the constructive remodeling of tissues and organs (Dahms et al., 1998; Meyer et al., 1998), with many of these ECM materials being marketed for a variety of therapeutic applications (Perniconi and Coletti, 2014; Teodori et al., 2014). Intact ECM is typically obtained by means of decellularization from explanted tissue in such a way as to create scaffolds that maintain the original spatial organization and biochemical composition. Tissue decellularization may be achieved in various ways, all of which eliminate the cellular compartment and leave a spatially and chemically preserved ECM (Crapo et al., 2011; Teodori et al., 2014).

We previously produced muscle acellular scaffolds (MAS) by means of decellularization at the whole organ scale of murine skeletal muscles. We characterized the *in vivo* response to grafted MAS and observed that such a construct provides a pro-myogenic environment (Perniconi et al., 2011). In particular, we reported that MAS orthotopically transplanted in mice was colonized by both inflammatory and stem cells and supported *de novo* muscle fiber formation (Perniconi et al., 2011). By definition MAS possesses only one component of the niche, i.e., the muscle ECM, being deprived of tissue specific cells and growth factors. In our previous experimental settings (Perniconi et al., 2011), as MAS was orthotopically grafted to replace a *Tibialis anterior* muscle (TA), the relative contribution to muscle formation by the graft and the surrounding environment could not be fully assessed because both MAS and TA were of muscular origin. The aim of the present work was to investigate whether MAS *per se* is exclusively a pro-myogenic environment (which would mean its use is limited to muscle tissue engineering applications) or is compatible with other differentiation pathways. In order to achieve this aim, we analyzed, *in vitro* and *in vivo*, the interaction between MAS and both muscle and non-muscle cells and tissues, to determine whether MAS is necessary or sufficient to induce myogenesis regardless of its location.

## Materials and methods

### Cell cultures

C2C12 mouse myoblasts were cultured in growth medium (GM), composed of Dulbecco's Modified Eagle Medium (DMEM) with 4.5 g/l Glucose, L-Glutamine (Sigma), supplemented with 15% fetal bovine serum (FBS), and 100 U/ml penicillin/100 microg/ml streptomycin (Invitrogen). For differentiation experiment positive controls, C2C12 were cultivated in GM until they reached 80–90% of confluence on plastic Petri dishes, then were shifted to 2% horse serum (HS) medium (DM). Alternatively, 2 × 10^6^ C2C12 cells were resuspended in 50 microL of GM and injected within a MAS derived from a murine TA (see the paragraph Decellularization of skeletal muscle, below). The cells were treated for 5–7 days with 2% HS (horse serum) in the absence or presence of BMP-2.

### Decellularization of skeletal muscle

For decellularization of skeletal muscle we dissected TA or *Extensor digitorum longus* (EDL) and immediately incubated them in sterile 1% SDS in distilled water for 48 and 24 h, respectively, at RT under slow rotation. At least 10 ml of SDS solution was used for each pair of muscles. After the decellularization procedure, the muscles were thoroughly washed by means of 3 incubations lasting at least 30 min each in sterile PBS. Decellularized scaffolds were used on the same day as they were produced or were stored for specific experiments.

### Animals and surgical procedures

Adult sex-matched BALB/C mice were used throughout this study as both donors and hosts. Mice were treated according to the guidelines of the Institutional Animal Care and Use Committee. Donor animals were sacrificed before skeletal muscle removal, while host animals were anaesthetized before muscle dissection and MAS engraftment. The transplantation procedure is described in detail below. TA acellular scaffolds were used to replace TA of inbred, age- and sex-matched wild type mice. The grafts were subsequently dissected from the host 2 weeks following transplantation. The surgical procedures have been described previously (Perniconi et al., 2011).

Scaffold grafting within the renal capsule. Animals were weighed and anaesthetized. After a small incision had been made in the body wall, the kidney popped out of the hole in the body wall when pressure was applied on either side of the kidney using the forefinger and thumb. An incision of approximately 5 mm was made in the renal capsule. Then a glass Pasteur pipette, which had been drawn thin and fire-polished with a rounded closed end, was used to obtain a capsule pocket by manipulating the pipette point tangential to the kidney in such a way as to detach the renal capsule. The scaffold was inserted into the pocket under the capsule using the polished glass pipette. After replacing the kidney within the peritoneal cavity, the body wall was sutured with 1 or 2 stitches of silk thread.

Scaffold grafting under the xiphoid process. Animals were weighed and anaesthetized. A small incision was made under the sternum. Without touching the muscle diaphragm, the scaffold was attached with a suture to the xiphoid process in order to suspend it within the peritoneal cavity in close contact with cartilage tissue. The body wall was sutured with 1 or 2 stitches of silk thread.

### Histological analysis

At the end of the experimental period the pellets within the scaffolds were frozen within OCT mounting medium (Leica) in liquid nitrogen-cooled isopentane. Cryosections (8 μm) were obtained using a Leica cryostat. For histological analysis, the sections were stained with hematoxylin and eosin using standard methods (Sigma). Alternatively, cryosections were stained with 0.05% Toluidine blue (BDH) for 30 min. Photomicrographs were obtained using an Axioscop 2 plus system equipped with an Axiocam HRc (Zeiss) at 1300 × 1030 pixel resolution.

### Alkaline phosphatase (ALP) assay for the characterization of osteoblastic phenotypes

A leukocyte ALP kit (Sigma-Aldrich) was used for ALP staining according to the recommended protocol. Cells were counterstained with 0.05% neutral red (Sigma). They were then fixed by immersion in fixative solution (citrate-acetone-formaldehyde) for 30 s, gently rinsed with water and put in an alkaline-dye mixture (sodium nitrite solution, FRV-Alkaline solution, deionized water, Naphthol AS-BI Alkaline solution) for 15 min. The samples were then rinsed for 2 min in deionized water and counterstained for 2 min with Hematoxylin solution Gill N.3, rinsed in tap water and air dried.

### Immunofluorescence analysis

Transverse cryosections were rinsed in PBS for 5 min at RT and then incubated with Blocking minBuffer (1%BSA, 10% Goat Serum in PBS) for 1 h at RT. The samples were washed in PBS and incubated with primary antibody (Ab) MF20 (Mouse IgG2b myosin Hybridoma bank) at a 1:50 dilution in PBS, and polyclonal anti-laminin Ab (policlonal Rabbit Sigma) at a 1:50 dilution in PBS. The sample were incubated with the secondary Ab anti-mouse-Dylight 549 and anti rabbit AlexaFluor 488 a 1:400 dilution in PBS for 1 h. Alternatively, laminin Ab was detected by anti-rabbit-Alexa 568 Ab. Secondary Abs were used to detect endogenous IgG on cryosections of the grafted material using anti-mouse-Alexa 488. Pre-immune serum was used for the negative control. Finally, 0.5 ug/ml Hoechst 33342 (Sigma) was used to counterstain cell nuclei. Photomicrographs were obtained by means of an Axioskop 2 plus system (Zeiss) or a Leica Leitz DMRB microscope fitted with a DFC300FX camera for confocal analysis (Leica).

Quantitative analysis was performed on fluorescence images of 10 randomly chosen microscopic fields acquired in the red channel. Post-processing of the images was performed using Adobe Photoshop and Scion Image Softwares (the latter, a software originally developed with the name of NIH Image at NIH, Bethesda, is freely downloadable at http://www.scioncorp.com/pages/scion_image_windows.htm). Modifications were the same for all images and consisted in a conversion to gray scale, followed by the measure of the mean fluorescence calculated on the whole field. The values were normalized by the area examined and expressed as arbitrary units (AU). Values deriving from the 10 sampled fields were averaged and the result was considered representative of one sample. At least four independent replicates were analyzed and averaged.

### Quantitative real-time reverse transcription polymerase chain reaction (RT-qPCR)

Total mRNA was isolated using an RNeasy mini kit (Qiagen, Courtaboeuf, France) or a TRIzol^®^ reagent method (Invitrogen) as described by the manufacturers. DNAse-treated RNA was reverse transcribed with SuperScript^®^ III RT (Invitrogen), the cDNA obtained was amplified using TaqMan^®^ or SYBR^®^Green chemistries (Applied Biosystems by Life Technologies, Courtaboeuf, France), and monitored with the 7500HT Fast Real-Time PCR System (Applied Biosystems). Primers used for RT-qPCR are listed below. Amounts of cDNA of interest were normalized to that of GAPDH (ΔCt = Ct gene of interest—Ct GAPDH). Results were reported as relative gene expression (2-ΔCt). The following couples of primers were used for murine (m) or human (h) genes: (1) glyceraldehyde 3-phosphate dehydrogenase (mGAPDH: forward CTGAGCAAGAGAGGCCCTA; reverse TATGGGGGTCTGGGATGGAA; (2) runt-related transcription factor 2 (mRUNX2: forward TTGACCTTTGTCCCAATGC; reverse AGGTTGGAGGCACACATAGG); (3) alkaline phosphatase (mALP: forward TGTCTGGAACCGCACTGAACT; reverse CAGTCAGGTTGTTCCGATTCAA); (4) glyceraldehyde 3-phosphate dehydrogenase (GAPDH: forward CCAGCAAGAGCACAAGAGGA; reverse AGATTCAGTGTGGTGGGGG); (5) runt-related transcription factor 2 (hRUNX2: forward GAATCCTCCACCCACCCAAG; reverse AATGCTGGGTGGCCTACAAA); (6) bone sialoprotein 2 (hIBSP: forward CCATTCTGGCTTTGCATCCG; reverse GACAAGAAGCCTATTACTTTGC); (7) bone sialoprotein 2 (hOC: forward GTGCAGAGTCCAGCAAAGGT; reverse TCCCAGCCATTGATACAGGT); (8) alkaline phosphatase (hALP: forward GTGCAGAGTCCAGCAAAGGT; reverse TCCCAGCCATTGATACAGGT); (9) myogenin (forward GCACTGGAGTTCGGTCCCAA; reverse TATCCTCCACCGTGATGCTG); (10) Myo D (forward ACCCAGGAACTGGGATATGGA; reverse AAGTCGTCTGCTGTCTCAAA); (11) embryonic myosin heavy chain (e-MHC: forward CGTCTGCTTTTGGCAA; reverse TGGTCGTAATCAGCAGCA). RUNX2 and ALP represent classic markers of osteogenic differentiation (Coquelin et al., 2012; Leotot et al., 2013), while MyoD, myogenin and myosin represent the standard markers for myogenic differentiation (De Arcangelis et al., 2003, 2005; Naro et al., 2003; Musaro et al., 2007).

## Results

### MAS is suitable for 3D myogenic cell cultures

**Figure 1:**
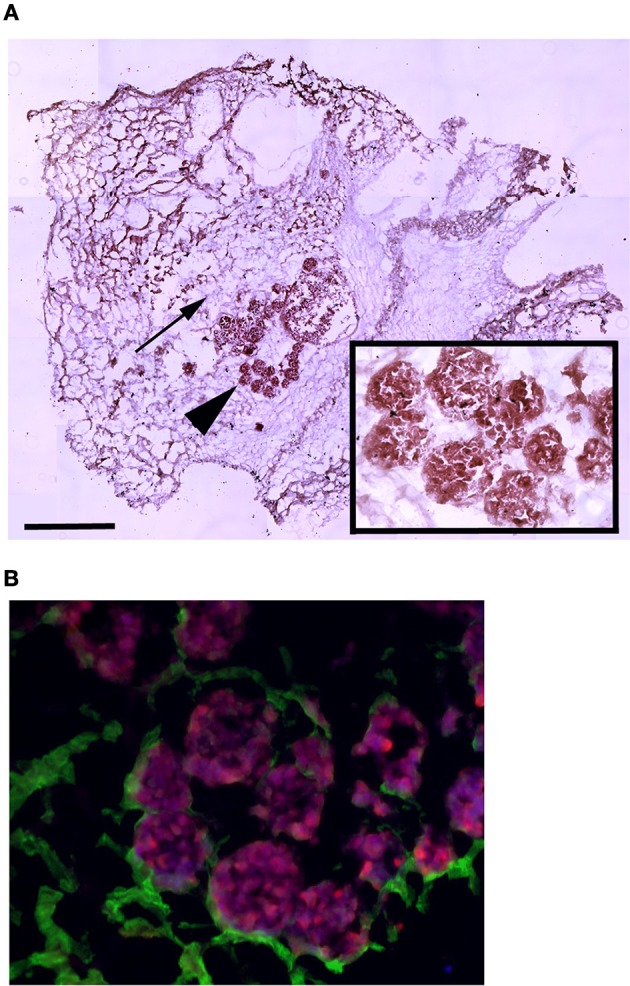
**MAS is suitable for 3D myogenic cultures *in vitro*. (A)** Hematoxylin and eosin staining of C2C12 cells cultured in MAS in myogenic differentiation medium for 1 week, showing both the sponge-like MAS network (arrow) and cell aggregates (arrowhead); the latter are also visible at a higher magnification in the inset. **(B)** Immunofluorescence localization of laminin (green) and myosin (red) on a serial section demonstrates that well differentiated muscle cells lie in aggregates on base membrane-derived laminin sheets. The image is representative of a triplicate experiment. Bar: 200 μ.

To assess whether MAS supports cell attachment, survival and differentiation, we cultured myogenic C2C12 cells in MAS in DM for 1 week. C2C12 is a multi-potent cell line derived from muscle satellite cells, and is thus primarily myogenic. On the other hand, MAS is a muscle-derived biomaterial which we had previously demonstrated is pro-myogenic *in vivo*, without knowing whether it retained the same properties *in vitro*. In these conditions, C2C12 cells showed the tendency to aggregate in a cell-cell fashion (Figure 1A), even though they clearly came into contact with the MAS laminin (Figure 1B), when cultured within MAS. The cells survived for several days in culture and were able to differentiate, i.e., to express markers of fully differentiated muscle fibers (Figure 1B). Therefore, we considered that MAS was sufficiently stable to support cell cultures and was compatible with myogenic differentiation.

**Figure 2:**
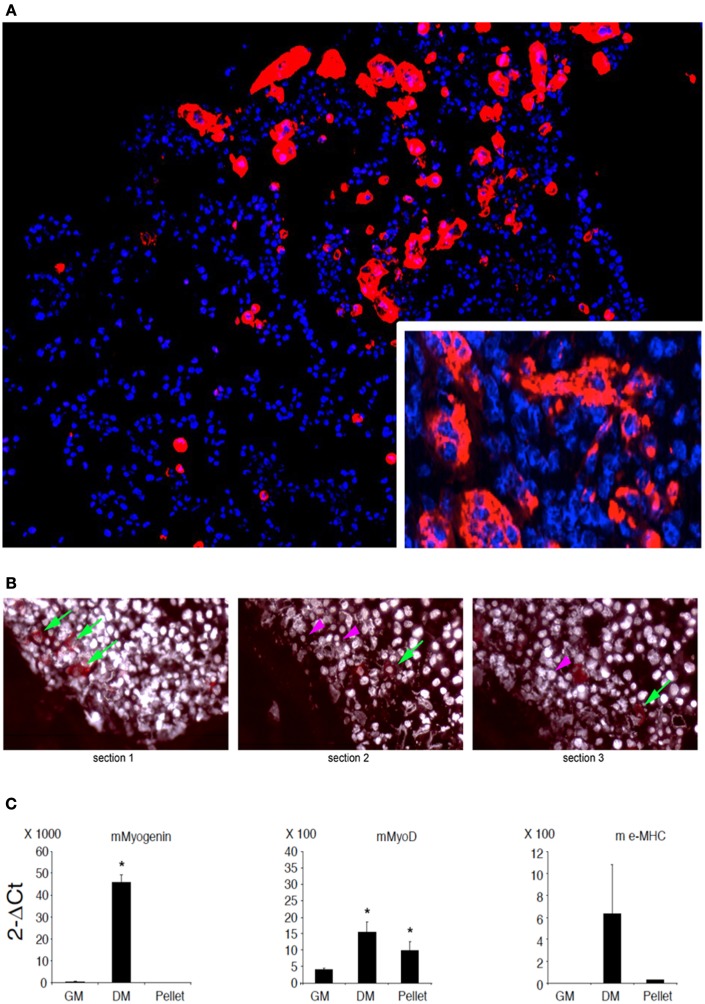
**C2C12 3D culture myogenic differentiation. (A)** Immunofluorescence analysis of all myosin expression (MF20 antibody, red) in pellets of C2C12, whose nuclei are counterstained by Hoechst (blue), cultured for 5 days in a minimal medium consisting of DMEM supplemented with 1% BSA. The inset at a higher magnification shows myosin positive syncytia. **(B)** Serial sections of C2C12 pellets (distance between section = 15 μm) following immunofluorescence analysis for myosin showing the appearance (green arrows) and disappearance (purple arrowheads) of the signal, suggesting that the dimension of the objects is of the order of magnitude of 30 μm. Nuclei are counterstained by Hoechst and post-processed to withe to ameliorate contrast. **(C)** Q-PCR of muscle markers expressed by C2C12 pellets cultured in 1% BSA for 5 days (pellet) as compared to C2C12 2D cultures in growth medium (GM) and differentiation medium (DM), used as a negative and positive control, respectively. From left to right: murine (m) myogenin, MyoD and embrionic (e) myosin heavy chain (MHC). The mean ± s.e.m. of triplicate samples is shown. ^*^p < 0.05 vs. GM, by Student's *t*-test. Culturing myogenic cells in 3D aggregates is sufficient to trigger a certain extent of myogenic differentiation. Therefore, whether MAS promotes muscle differentiation in serum-free medium (i.e., in the absence of pro-myogenic stimuli) cannot be clearly ascertained due to lack of proper 3D culture controls.

C2C12 are cells that fuse with each other during differentiation to form myotubes *in vitro*. As expected, cell-cell contact represents a condition leading to fusion for these cells (Rochlin et al., 2010). However, this is not a sufficient trigger when C21C2 are cultured in a minimal medium supplemented with 1% BSA in 2D cultures, a condition that is not associated with their myogenic differentiation and cell fusion. On the other hand, 3D cultures of C2C12 are still poorly characterized (Carosio et al., 2013). Therefore, we decided to characterize the effects of 3D culturing *per se* on C2C12 differentiation with the aim to better interpret the C2C12 3D cultures within MAS. We noticed that 3D C2C12 aggregates obtained by pelleting C2C12 before placing them in culture, displayed several markers of myogenic differentiation both in 2% HS in 1% BSA (Figure 2 and data not shown). In particular, C2C12 2D cultures in 2% HS (the gold standard myogenic differentiation for this type of cells) were compared to C2C12 pellets cultured in 1% BSA. The latter showed: not detectable myogenin but significant MyoD expression (Figure 2C), which was sufficient to trigger myosin expression (detectable with an anti-pan-myosin antibody such as MF20, Figure 2A). Among the myosin isoforms possibly expressed by C2C12, we found low embryonic myosin expression (Figure 2C), suggesting that other isoforms typical of more mature fibers are expressed, in spite of the absence of elongated myotube formation (Figure 2B). Such aberrant differentiation resembled that of rhabdomyosarcomas and the Rb cell line (Wang et al., 2010). However, since a 3D organization was *per se* myogenic to a certain extent, a 3D C2C12 culture in MAS in 1% BSA was considered to be unsuitable to test whether MAS was sufficient to induce differentiation under minimal medium, conditions owing to the lack of an adequate negative control *in vitro*.

### MAS does not irreversibly induce 3D cell cultures to a myogenic fate

**Figure 3:**
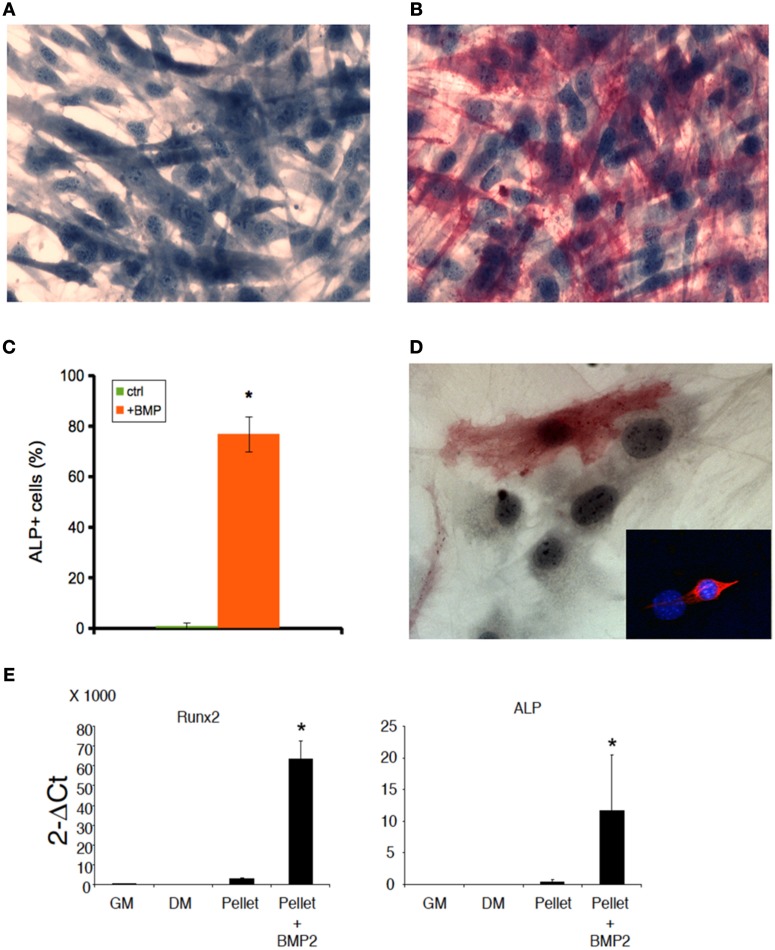
**BMP induces osteogenic differentiation both in 2D and 3D C2C12 and primary cultures**. ALP staining of C2C12 cultures on plastic in the absence **(A)** or presence **(B)** of 300 ng/ml BMP-2 for 5 days, showing that C2C12 possess osteogenic potential when cultured in the presence of BMP. The cells were counterstained with Hematoxylin and the osteoblasts (red cells) were quantified **(C)**. The mean ± s.e.m. of quadruplicate samples is shown. ^*^p < 0.05 vs. GM, by Student's *t*-test. **(D)** A similar myogenic to osteogenic conversion upon BMP treatment occurs in myogenic cell primary cultures: an ALP+ cell is visible in the photomicrograph; the inset shows the myogenic lineage of primary cultures from skeletal muscle by desmin expression (red), while nuclei were counterstained by Hoechst (blue). Nuclear size and shape discriminate myoblasts/osteoblasts from fibroblasts. Bar: 50 μ. **(E)** Q-PCR of bone markers expressed by C2C12 pellets cultured in 1% BSA for 5 days (pellet), in the absence of presence (+BMP-2) of 300 ng/ml BMP-2, as compared to C2C12 2D cultures in growth medium (GM) and differentiation medium (DM), both used as a negative controls for osteogenic markers. From left to right: murine runt-related transcription factor 2 (Runx2) and alkaline phosphatase (ALP). The mean ± s.e.m. of triplicate samples is shown. ^*^p < 0.05 vs. GM, by Student's *t*-test.

Both C2C12 cells and muscle primary cell cultures, while committed to a myogenic lineage, differentiate into osteoblasts in the presence of BMP (Katagiri et al., 1994; Friedrichs et al., 2011). We confirmed this notion for 2D C2C12 and primary satellite cells obtained from murine skeletal muscle and cultured for 5 days in 2%HS supplemented with 300 ng/ml BMP-2 (Figures 3A–D). BMP-treated cells displayed loss of myogenic differentiation, such as multinucleated myotube formation (Figure 3A) in favor of a potent pro-osteogenic conversion as demonstrated by the presence of non-fused, ALP expressing cells (Figures 3B–D). Worth noting BMP-2 effects on standard osteogenic markers (Coquelin et al., 2012; Leotot et al., 2013) were still potent in 3D C2C12 cultures (Figure 3E), indicating that 3D cultures are not impermeable nor insensitive to BMP treatment and suggesting to use BMP as a non-myogenic cue to test whether MAS irreversibly commit C2C12 to a non-myogenic fate.

**Figure 4:**
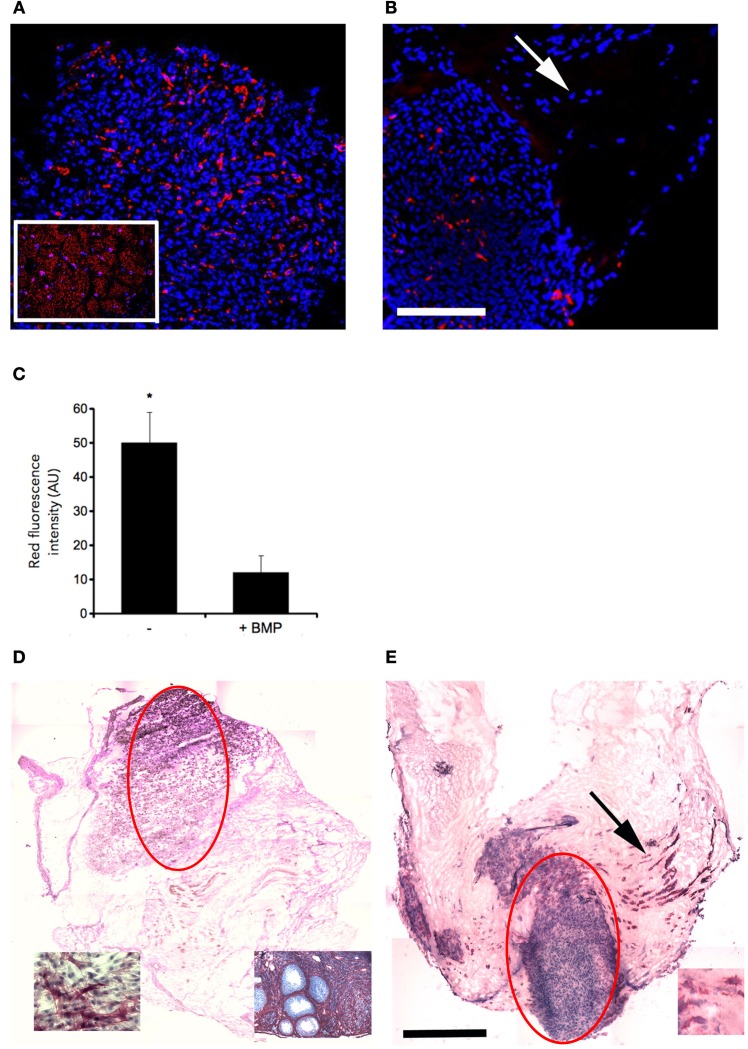
**MAS does not irreversibly commit C2C12 cells to a myogenic fate**. Immunofluorescence analysis of all myosin expression (MF20 antibody, red) in C2C12 cells, whose nuclei are counterstained by Hoechst (blue), cultured for 5 days in MAS in a medium consisting of DMEM supplemented with 2% HS in the absence **(A)** or presence **(B)** of 300 ng/ml BMP-2. The inset in **(A)** shows a muscle section used as a positive control. Myosin positive cells (red) are visible in both cases; however, myogenic differentiation seems to occur predominantly in tightly aggregated cells, since the scattered, ALP positive cells do not express myosin in serial sections (see the arrows in **B,E**). **(C)** Quantification of myosin (red fluorescence) from quadruplicate samples as above. Mean ± s.e.m. ^*^p < 0.05 vs. GM, by Student's *t*-test. As shown by ALP staining, and hematoxylin counterstaining, of C2C12 cells cultured in MAS in the absence **(D)** or presence **(E)** of BMP, C2C12 do express ALP but only in the presence of BMP. The bulk cell aggregates deriving from the original injection of cell suspension into the MAS are indicated by light ellipses. The insets in **(D)** represent positive controls: an ovary section (left) and BMP-treated C2C12 cells (right); the inset in **(E)** shows ALP expressing cells at a higher magnification. The latter are abundant in the region in which MAS was colonized by cells (arrow in **E**), which are likely to have migrated out of the cell aggregate visible in the center of the MAS. Black bar = 500 μ, white bar = 100 μ.

To this purpose, we treated C2C12 cultured in MAS for 5 days within 2%HS in the absence or presence of 300 ng/ml BMP-2. While C212 in MAS accumulated myosin and did not express ALP (Figures 4A,D), the same cells showed significantly reduced myosin expression and differentiated in ALP-expressing osteoblasts in the presence of BMP (Figures 4B,C,E). Interestingly, ALP expression was stronger in those cells that displayed migratory activity by leaving the bulk cell aggregate and becoming isolated cells (Figure 4E, inset). Thus, MAS supports at least two differentiation pathways for cells of mesenchymal origin and, while participating in the muscle environment *in vivo*, does not suppress the pro-osteogenic stimulus induced by BMP-2 *in vitro*.

### MAS promotes muscle regeneration in volumetric muscle loss

**Figure 5:**
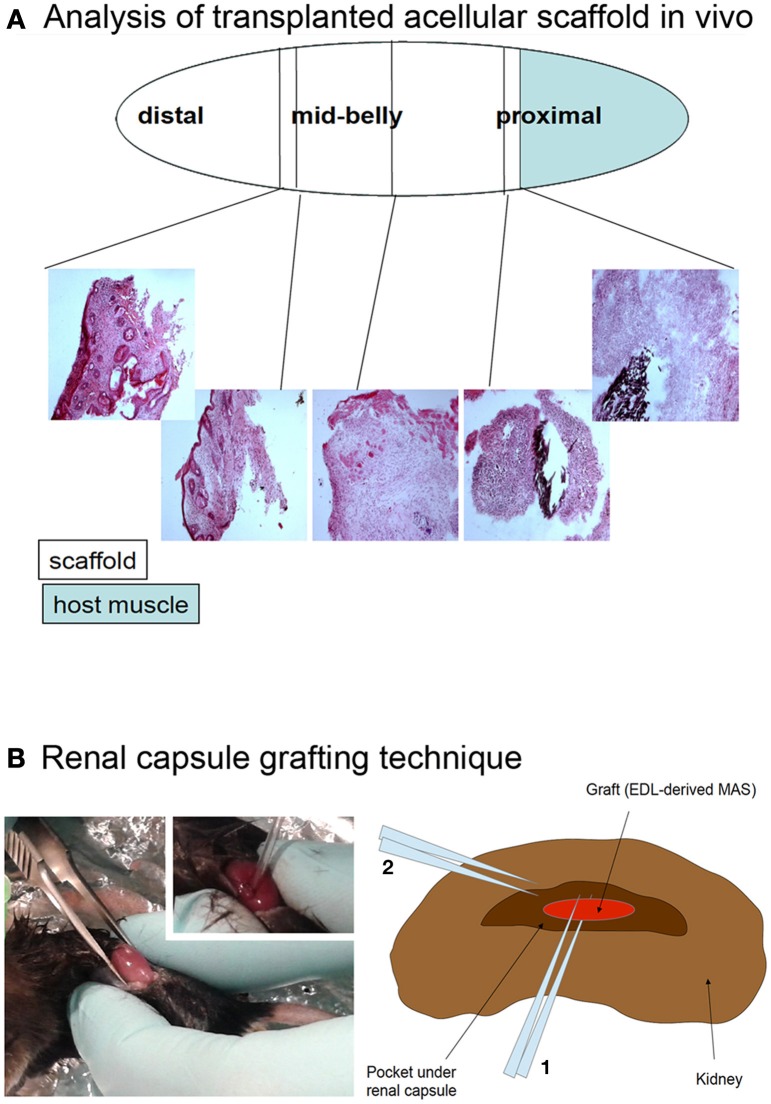
**(A)** Analysis of transplanted acellular scaffold *in vivo*. The *Tibialis anterior* muscle was removed, with the exception of a proximal fragment (light blue) and the MAS (open ellipse) was sutured to the muscle at the proximal end and to the tendon at the distal end. The black silk suture knots, visible in the hematoxylin and eosin stained cryosections, were used as a reference of the graft extremities. The first third, second third and last third of the graft, starting from the proximal region, were defined as the proximal, mid-belly and distal parts of the grafted MAS, respectively. **(B)** Renal capsule grafting technique. The kidney was exposed by dorsal skin incision (picture) and the renal capsule cut lengthwise for about 5 mm; a pocket was created under the connective tissue renal capsule by using a fire polished Pasteur pipet (picture inset). The graft was inserted into the pocket by mean of forceps and the tip of the pipet (2 and 1 in the drawing, respectively). For this specific experiment, the grafted MAS was obtained from an *extensor digitorum longus* muscle (*Graft, EDL-derived MAS*) because of the size of the latter was compatible with the grafting technique.

To assess *in vivo* whether MAS itself is inherently myogenic, we grafted the MAS both orthotopically (i.e., replacing a *Tibialis anterior*) and ectopically (i.e., within the renal capsule or underneath the xiphoid process). The Figure 5 illustrates these procedures, that are described in detail elsewhere and represent standard *in vivo* approaches to test xeno- or auto-grafts (Mericskay et al., 2004; Perniconi et al., 2011).

**Figure 6:**
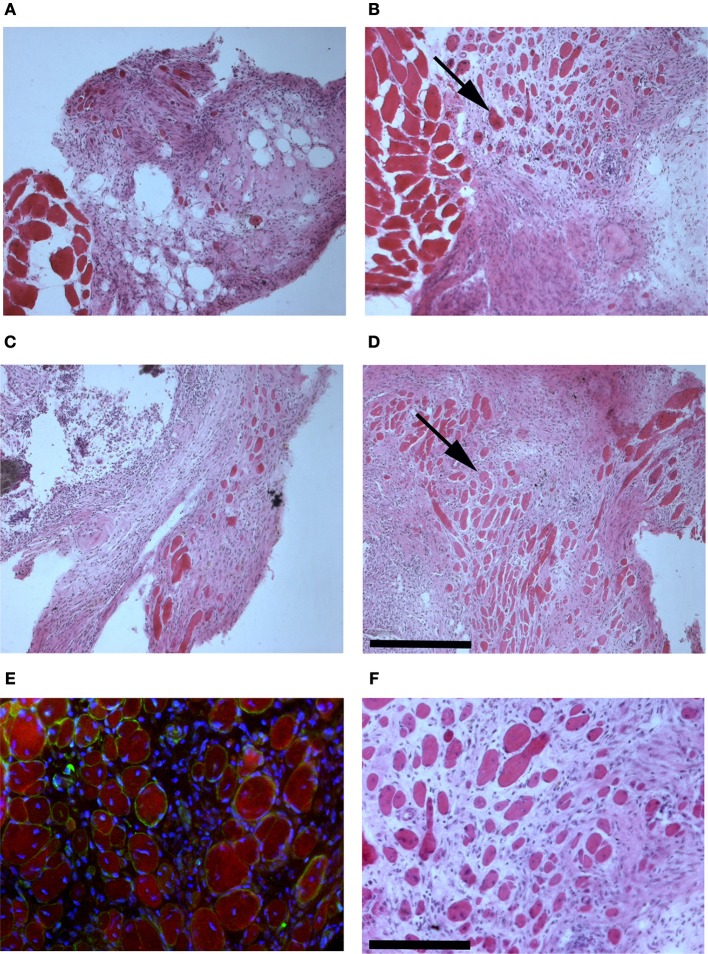
**MAS is sufficient to support muscle regeneration in volumetric muscle loss**. Hematoxylin and eosin staining of cross-cryosections of MAS grafted in the place of the *Tibialis anterior* in a syngeneic mouse and analyzed at the level of distal **(A,C)** and mid-belly **(B,D,F)** muscle sections (for the definition of these positions, refer to Figure 5A), 2 **(A,B)** and 3 **(C,D,F)** weeks following transplantation. Numerous regenerating muscle fibers (arrow), characterized by centrally located nuclei, are visible within the inflammatory infiltrate. Multiple and/or centrally located nuclei in the same rose cytoplasm indicate bona fide nascent muscle fibers **(F)**, as confirmed by sarcoglycan (green) and myosin (red) expression around centrally located nuclei (blue) shown by immunofluorescence in **(E)**. The picture is representative of triplicate experiments. Bar = 200 μ.

**Figure 7:**
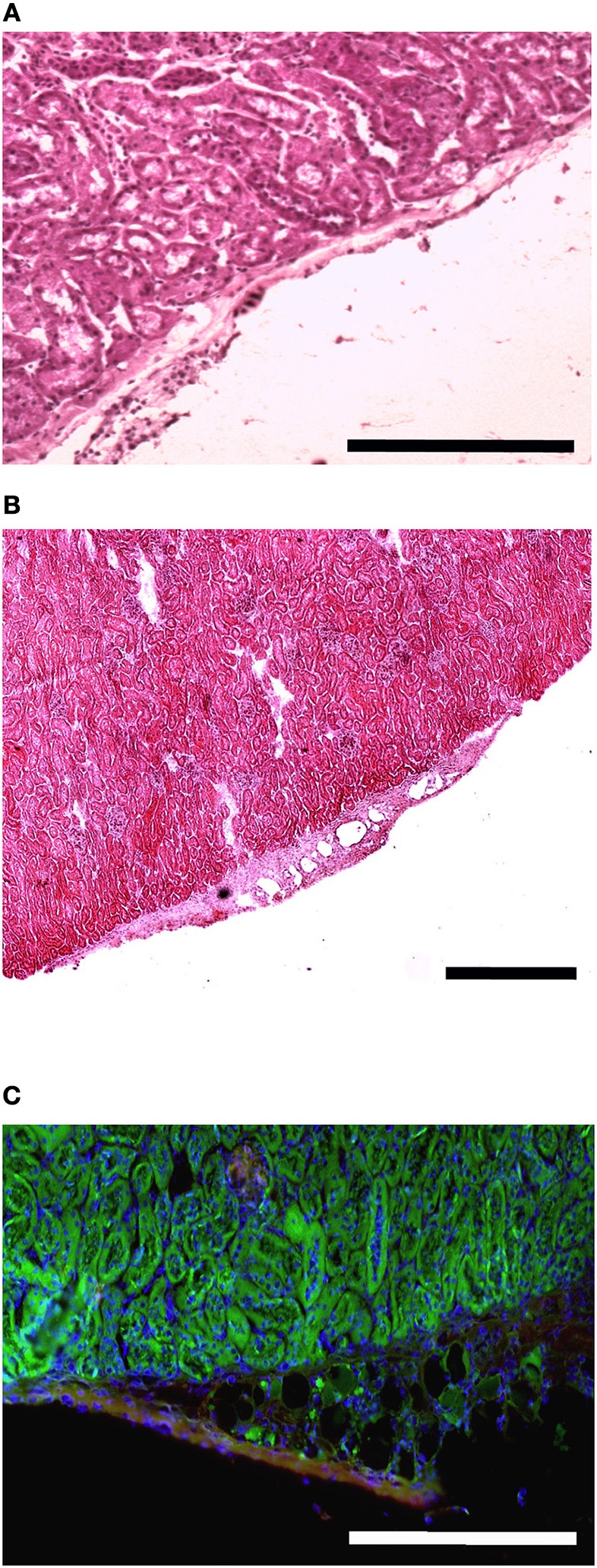
**MAS is not sufficient to promote myogenesis within the renal capsule**. Hematoxylin and eosin staining of cross-cryosections of a peripheral region of the kidney and its renal capsule in the absence **(A)** or in the presence **(B)** of grafted MAS, 2 weeks following transplantation. The immunofluorescence analysis **(C)** for laminin (green) and myosin (red) shows the absence of muscle fibers in the grafted MAS. Note that the glomeruli are strongly autofluorescent. Nuclei are stained by Hoechst (blue). Bar = 200 μ. The picture is representative of triplicate experiments.

The presence of muscle fiber formation within orthotopically grafted MAS (Figures 6A–D) confirmed our previous *in vivo* results (Perniconi et al., 2011). Within 2 weeks from transplantation MAS was colonized by cells and showed the presence of nascent muscle fibers characterized by centrally located nuclei and muscle fiber specific markers such as myosin and sarcoglycan (Figures 6E,F), thereby demonstrating that MAS represents an environment that is compatible with neo-myogenesis. We found it striking that MAS alone, i.e., an empty scaffold even though of muscle origin, was colonized by cells and hosted new muscle formation, suggesting that it represents a pro-myogenic niche *per se*. To verify whether MAS was sufficient to induce myogenesis, we grafted MAS in anatomically different regions, i.e., the renal capsule and the peritoneal cavity under the xiphoid process. The renal capsule represents the gold standard for *in vivo* transplantation and assessment of allografts survival, bioactivity and function, because it is a highly vascularized environment that lends itself to the rapid integration and nutrition of the grafts. Two weeks following transplantation, we found grafted MAS (derived from EDL muscle for this specific experiment) within the renal capsule, which retained its sponge-like, laminin-based structure. While we observed that numerous cells came into contact with and colonized MAS, we did not detect any overt differentiated muscle fiber, by either the morphological or immunofluorescence analysis of muscle fibers (Figure 7).

**Figure 8:**
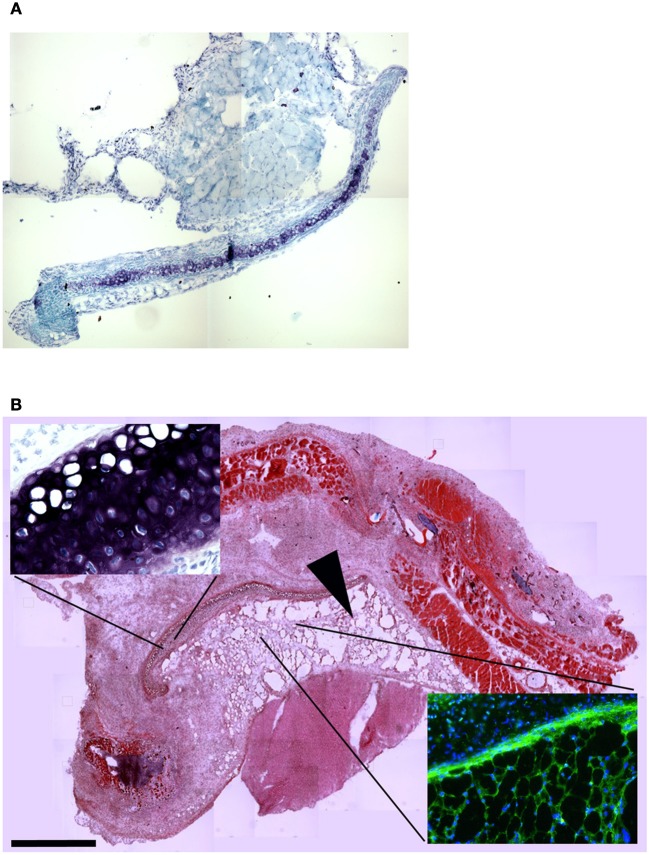
**MAS grafted under the xiphoid process. (A)** Toluidine blue staining of the region of the xiphoid process without grafting. Cartilage tissue (arrow) is strongly metachromatic. **(B)** Hematoxylin and eosin staining of the same region including grafted MAS, 2 weeks following transplantation. The absence of muscle fibers in the grafted MAS (arrowhead) is evident when the latter is compared with skeletal muscles of the pectoral muscles in the upper region of the image. The insets, at a higher magnification, show: (bottom right) immunofluorescence analysis for laminin (green) and myosin (red)—nuclei are stained by Hoechst (blue)—on a serial section demonstrating the absence of muscle fibers in the grafted MAS; (upper left) toluidine blue staining showing the position of the xiphoid process, whose matrix is highly metachromatic. The picture is representative of triplicate experiments.

**Figure 9:**
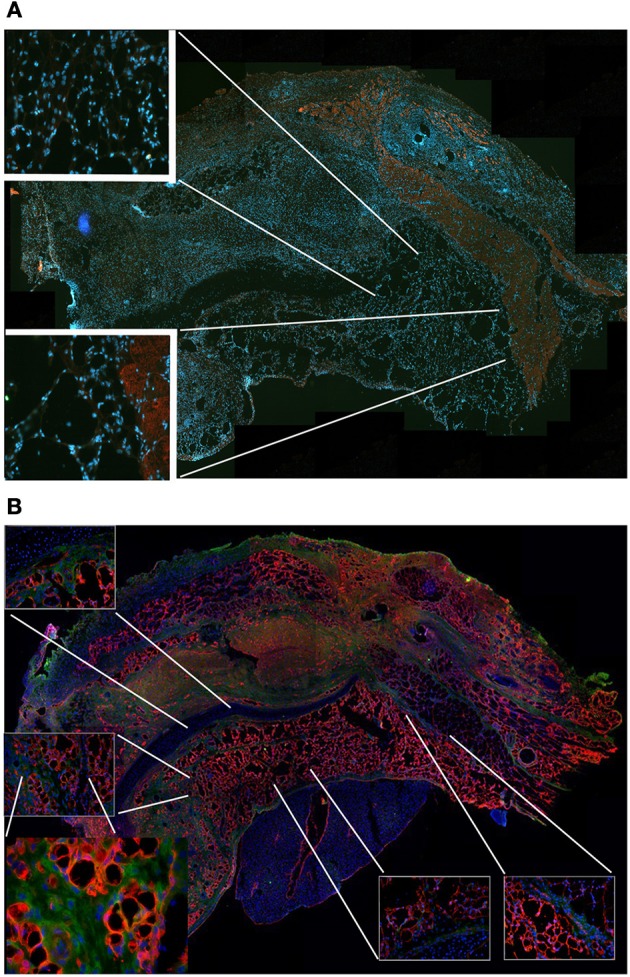
**MAS is not sufficient to promote myogenesis under the xiphoid process. (A)** The panel represents a reconstruction (with insets at a higher magnification) of the whole region, following an immunofluorescence analysis for myosin (red) and nuclei (blue), once again demonstrating the absence of muscle markers in the spongy structure of MAS; this is in contrast to the pectoral muscles in the upper region of the image, which represent a positive control for the immunofluorescence reaction. **(B)** Reconstruction (with insets at a higher magnification) of the whole region, following an immunofluorescence analysis for laminin (red), immunoglobulins (green) and nuclei (blue), which demonstrates MAS colonization by cells during an inflammatory process. All the analyses in this figure were performed 2 weeks following transplantation, which is a sufficient amount of time for muscle regeneration in MAS grafted to replace a *Tibialis* muscle. The picture is representative of triplicate experiments. Bar = 400 μ.

To confirm this result, we grafted MAS under the xiphoid process by suturing it to the latter. In physiological conditions under the xiphoid process, which is a cartilaginous organ enveloped by connective tissue, there is the empty intraperitoneal space and the liver (Figure 8A). When grafted in this position, MAS was recognized by its sponge-like structure but could not be confirmed as a site of neo-myogensis, even though many cells did colonized the MAS and interacted with its laminin matrix (Figure 8B). Most of the cells migrating within the MAS and along the laminin are, in fact, likely to have been inflammatory cells, as demonstrated by the presence of abundant immunoglobulins (Figure 9A), which is consistent with our previously published findings. As a result, while myosin positive muscle tissue is visible in the analyzed sections, this is clearly not deriving from the grafted scaffold, on the basis of its histology and anatomical localization, which is distant and not in continuity with the grafted MAS (Figure 9B).

All together, the aforementioned results indicate that MAS *per se* is not sufficient to induce myogenesis *in vivo*, but it requires the presence of surrounding muscles to show robust myogenesis, an effect further increased if MAS is seeded with myogenic cells before engraftment.

## Discussion

In tissue engineering interventions, grafting of decellularized tissues or organs, such as MAS, is an increasingly widespread approach in pre-clinical and clinical settings (Badylak et al., 1998, 2013; Perniconi and Coletti, 2014; Teodori et al., 2014). In particular, ECM of muscle origin has been proposed as en efficient scaffold for organ-scale reconstruction following volumetric muscle loss (Ott et al., 2008; Perniconi et al., 2011). When MAS is orthotopically grafted in mouse to replace a skeletal muscle, *de novo* myogenesis is observed within a few weeks, probably as a result of MAS colonization by stem cells of host origin with myogenic potential (Perniconi et al., 2011). However, the relative contribution to muscle formation by the graft and the surrounding environment cannot be fully understood using this approach, because both are of muscular origin. In order to verify whether MAS possesses pro-myogenic properties *per se*, i.e., regardless of the site of transplantation, we grafted MAS in a heterotopic position in syngeneic mice. Using this approach, here we demonstrate that MAS is neither sufficient nor necessary for myogenesis. Indeed, when MAS is transplanted within the renal capsule or the peritoneal cavity under the xiphoid process it is colonized by an abundant cell infiltrate but does not display any regenerating fiber within its laminin network. These results demonstrate that MAS is stable in anatomical sites other than the skeletal musculature, but does not provide enough signals to trigger myogenesis by the colonizing cells. By cultivating C2C12 cells in 3D aggregates, we showed that the 3D culture condition is a potent pro-myogenic cue, that facilitates the formation of fully differentiated myotubes, which is in agreement with other reports (Carosio et al., 2013); 3D cell-cell contacts bypass inhibitory signals for muscle differentiation, such as culture in minimal, serum-free medium (Minotti et al., 1998; De Arcangelis et al., 2003), and promote the formation of a functionally active construct capable of contraction. From these results, we conclude that MAS is not necessary for muscle formation in 3D cultures. We do, however, confirm the results of our previous study (Perniconi et al., 2011), demonstrating that MAS is an excellent support for 3D myogenic cultures, both *in vitro* and *in vivo* (in the latter case, when orthotopically grafted). In addition, MAS is compatible with the differentiation toward the osteogenic lineage, obtained by BMP-2 treatment of the C2C12 cell line, which thus demonstrates that MAS does not represent a signal that fully and irreversibly commits cells to a myogenic fate. In conclusion, MAS has scaffold properties insofar as it supports cell attachment, migration, survival and differentiation. Whether MAS is a good support for cells of non-muscle origin and whether it can be exploited in trans-species experiments, such as cultivating human cells in murine MAS, remains to be addressed. However, accumulating evidence suggests that acellular scaffolds of biological origin are multipurpose and may be exploited for cell culture and tissue engineering of different tissue types regardless of their origin (Badylak et al., 1998; Conconi et al., 2005; Wolf et al., 2012).

It is widely accepted that the niche supports stem cells and controls their self-renewal *in vivo* (Spradling et al., 2001) by modulating asymmetric cell division and ensuring stem cell renewal and the production of a number of committed daughter cells that is sufficient for tissue homeostasis and repair (Kuang et al., 2008). The notion of the niche appears to be closely linked to that of the stem cell; however, in addition to affecting stem cell renewal, the microenvironment also controls commitment and differentiation of daughter cells deriving from asymmetrically dividing stem cells (Zhang et al., 2009; Bhattacharyya et al., 2012). Intriguingly, the niche itself is not always indispensable for asymmetric division and stem cell renewal, as shown by the case of lymphocyte differentiation in the absence of a permanent niche (Chang and Reiner, 2008). Cell-intrinsic factors, such as DNA strand asymmetric division and segregation, play additional, important roles in determining the asymmetric fate of daughter cells (Shinin et al., 2006). A very recent report established a novel paradigm for stem-cell maintenance according to which a dynamically heterogeneous cell population functions long term as a single stem-cell pool (Ritsma et al., 2014). Our findings suggest that this degree of plasticity might be extended to committed cells and be inherent to ECM components.

The notion of the microenvironment is of paramount importance when dealing with tissue engineered constructs aimed at regenerative medicine applications. Although some features of regeneration are shared by different tissues whereas others are tissue-specific, all those features are ultimately controlled by the tissue microenvironment. Understanding the mechanisms underlying tissue regeneration, particularly the choice between tissue restoration and reconstruction (two different strategies following tissue injury), is essential for regenerative medicine interventions (Coletti et al., 2013). The microenvironment (including the stem cell niche) is determined by three major components: the ECM, the cells and the local growth factors. In our work, we investigated whether MAS (which is basically composed of muscle ECM) is sufficient to recapitulate the effects of the muscle microenvironment on cell differentiation. We found that MAS displays features that are typical of a scaffold, though it is not strictly limited to muscle applications since it does not irreversibly commit cells to a myogenic fate. We conclude by saying that since MAS does not fully recapitulate muscle-specific microenvironment properties, it may be more plastic, and consequently easier to exploit, than has previously been believed.

### Conflict of interest statement

The authors declare that the research was conducted in the absence of any commercial or financial relationships that could be construed as a potential conflict of interest.
